# Cross-reactivity between antibodies induced by European swine influenza vaccines and Spanish swine influenza strains

**DOI:** 10.3389/fvets.2025.1725563

**Published:** 2026-02-04

**Authors:** Paloma Encinas, Aitor Nogales, Martha I. Nelson, Adolfo García-Sastre, Gustavo del Real

**Affiliations:** 1 Department of Biotechnology, National Institute for Agricultural and Food Research and Technology (INIA-CSIC), Madrid, Spain; 2 Department of Animal Health. Faculty of Veterinary Medicine, Complutense University of Madrid, Madrid, Spain; 3 Animal Health Research Center (CISA), National Institute for Agricultural and Food Research and Technology (INIA-CSIC), Madrid, Spain; 4 Division of Intramural Research, National Library of Medicine, National Institutes of Health, Bethesda, MD, United States; 5 Department of Microbiology, Icahn School of Medicine at Mount Sinai, New York, NY, United States; 6 Global Health and Emerging Pathogens Institute, Icahn School of Medicine at Mount Sinai, New York, NY, United States; 7 Division of Infectious Diseases, Department of Medicine, Icahn School of Medicine at Mount Sinai, New York, NY, United States; 8 The Tisch Cancer Institute, Icahn School of Medicine at Mount Sinai, New York, NY, United States; 9 Department of Pathology, Molecular and Cell-Based Medicine, Icahn School of Medicine at Mount Sinai, New York, NY, United States; 10 The Icahn Genomics Institute, Icahn School of Medicine at Mount Sinai, New York, NY, United States

**Keywords:** swine, influenza, vaccine, public health, zoonoses

## Abstract

**Background:**

Vaccination is an essential tool for controlling the severity of influenza in swine and its spillover risk to humans. Due to fast mutation rates, genomic reassortment, and the high antigenic variability of influenza A viruses (IAV), it is necessary to update vaccine strains to better match and neutralize circulating viruses.

**Methods:**

In this study, we analyzed the immune cross-reactivity of hemagglutination inhibition (HI) antibodies induced by currently approved European swine influenza vaccines against Spanish swine influenza viruses (SIVs) isolated from 2016 to 2021, as well as human IAV strains, using a murine model. Sera from immunized mice with monovalent (MVV) or trivalent (TVV) swine influenza vaccines were tested against Spanish representative SIVs carrying HAs from different subtypes and lineages using a HI serological assay. Amino acids in the antigenic motifs of the receptor binding site (RBS) of the IAV HA were compared and contrasted among SIV strains to detect changes that may affect protein antigenicity.

**Results:**

Sera from mice immunized with MVV or TVV showed no HI antibodies against a 2019 Eurasian avian-like H1 (EAswH1) SIV. TVV did not induce cross-reactive HI antibodies against two human seasonal H3 SIV from the 2000s (2000s-like H3) or against the human seasonal H3 vaccine strain (HuVacH3). Geometric mean (GM) HI titers of sera from TVV-immunized mice were below the protection threshold (GM < 40) against the recent human seasonal-like H1 (HUswH1) SIV, against an EAswH1 SIV, and against the human seasonal H1 vaccine strain (HuVacH1). HI antibodies induced by MVV showed high cross-reactivity with a 2019 EAswH1 SIV isolate.

**Conclusion:**

Currently authorized vaccines do not induce HI antibodies against some contemporary SIV circulating in Spain, nor against human seasonal influenza vaccine strains. Updating vaccine strains to better match new SIVs emerging in Spanish swine is warranted.

## 1 Introduction

Influenza A virus (IAV) belongs to the *Orthomyxoviridae* family and is the cause of swine influenza, a respiratory illness endemic in most swine-producing countries around the world. Swine influenza has a high morbidity rate that can reach 100% and a low mortality rate in naïve pigs (1). Clinical signs typically manifest as fever, fatigue, sneezing, coughing, dyspnea, and decreased appetite, which are indicative of acute respiratory disease (2). The disease is responsible for large economic losses as a result of reproductive failure in sows and weight loss or decreased gain in piglets (3). Furthermore, other pathogens can co-infect with IAV within the Porcine Respiratory Disease Complex (PRDC), which can lead to higher rates of mortality (4). In addition, swine influenza viruses (SIVs) pose a zoonotic risk as they can infect humans (5), since both share the same viral receptor in the respiratory tract, the alpha (2,6)-linked sialic acid (6). Once human influenza viruses are introduced and adapted to pigs, they can spill back to humans and cause sporadic cases of human influenza variants with varying pathogenicity (7–9). In addition, SIVs can reassort with avian influenza viruses and produce new emerging strains capable of replicating and spreading to humans, with the risk of causing an influenza pandemic, like the one in 2009 (10).

Swine influenza is enzootic worldwide in different combinations of hemagglutinin 1 (H1), hemagglutinin 3 (H3), and neuraminidases 1 (N1) and 2 (N2). H1N1, H1N2, and H3N2 are the most frequent subtypes (11, 12). The H3N1 subtype, although rare globally in swine, was detected in Spanish white pigs in prior analysis (13). Within each subtype, different SIV lineages are found depending on the geographical region. Current H1 SIVs in Europe belong to three evolutionarily lineages: 1C Eurasian avian-like (EAswH1), 1B human seasonal-like (HUswH1), and 1A pandemic-like (pdmH1) following the Global Nomenclature System for SIVs (14). Regarding the H3 subtypes, two clades with a human origin (HUswH3) are currently present in Europe: human seasonal-like H3 from the 1970s (1970s-like H3) and novel human seasonal-like H3 from the 2000s (2000s-like H3) (11, 12, 14). We have previously described the genetic composition of field SIV isolates in Spain in the period 2015–2019 (13). That study revealed the high genetic and antigenic variability of SIVs present in pigs from Spain, one of the world’s leading porcine producers and exporters, as well as the high rate of genetic change of SIVs in the pig population and their capacity to generate new lineages that can be easily exported to other countries. Therefore, this heterogeneous virus collection should be closely monitored to reveal and control the potential emergence of new SIV with pathogenic risk for both pigs and humans.

Vaccination is the most important tool to reduce the disease burden of influenza in pigs from swine or human origin (15), and its efficacy depends mainly on the induction of neutralizing serum antibodies against viral hemagglutinin (HA), the main antigenic protein, which mediates virus entry into host cells, and to a lesser extent on neuraminidase (NA), which mediates the viral release from the cell (12). Amino acid substitutions in the antigenic motifs of the receptor binding site (RBS) of the HA may drive changes in antigenicity that decrease the grade of antibody recognition induced by vaccines (16). To provide optimal protection, vaccine strains should match circulating viruses as closely as possible (17). Human seasonal influenza vaccines are updated annually due to rapid antigenic drift from one season to the next; meanwhile, current commercial swine vaccines are not updated as frequently as necessary, despite the constant antigenic diversification of SIV (12). Therefore, there is a need for more protective vaccinations or immunization techniques due to the ever-growing diversity of SIV to protect both human and animal health.

Hemagglutination inhibition (HI) assays are used by the World Health Organization (WHO) as a measure of serological immunity in risk assessment tools for IAV infection (18), and HI titers above 40 are considered a correlate of protection against influenza infection (19).

In this study, we aim to measure cross-reactive HI antibodies induced by two commercially available swine influenza vaccines in the murine model against field Spanish SIV isolates and two human seasonal IAV strains.

## 2 Materials and methods

### 2.1 Swine vaccines

FluPan and Flu3 (Ceva Santé Animal, France) are licensed and commercially available in Europe as swine influenza vaccines. FluPan is a monovalent vaccine (MVV) containing 16 HA units of influenza A/Jena/VI5258/2009 (pdmH1N1) and FLU3 is a trivalent vaccine (TVV) which includes 10–12 log_2_ geometric mean of neutralizing units (GMNUs) of the following strains: A/Bakum/IDT1769/2003 (H3N2,1970s-likeH3), A/Haselünne/IDT2617/2003 (H1N1, EAswH1) and A/Bakum/1832/2000 (H1N2, HUswH1). No new swine influenza vaccine formulations are available in Europe.

### 2.2 Immunization of mice

Animals were housed under pathogen-free conditions at the animal facility of the Animal Health Research Center (CISA-INIA/CSIC), Madrid (Spain). Female Balb/c 5-weeks-age mice were divided into three groups (Mock, MVV, and TVV), 7 animals per group, and immunized intraperitoneally with two doses of 0.1 mL of vaccine/control separated by 2 weeks. The dose administered to mice was proportional in weight to that indicated for pigs by the manufacturer, and the injection schedule is the usual one in the mouse model (20). The mock group was administered with phosphate saline buffer (PBS). Three weeks after the boost dose of vaccine, the mice were euthanized intraperitoneally using a lethal dose of ketamine and xylazine. Then, blood collection was conducted via cardiac puncture.

### 2.3 Mice sera treatment and serological assay

HI assays were performed according to the WHO practices (21) using fresh turkey red blood cells. Individual serum samples were pre-treated with a receptor-destroying enzyme (Sigma-Merck, Germany) overnight, and the day after, heated at 56 °C for 30 min to inactivate the enzyme. Sera were adsorbed with turkey red blood cells for 1 h to avoid unspecific hemagglutination reactions. Sera were subjected to six serial 2-fold dilutions (1:20–1:640). HI assays were performed using “V” bottom microtiter plates (Nunc, Thermo Scientific). Results were considered positive if serum dilution was 40 or above inhibited hemagglutination.

We did not study the cellular immune response, which, although lower in the case of inactivated vaccines, may also provide some degree of cross-protection.

### 2.4 Viruses

**Figure 1:**
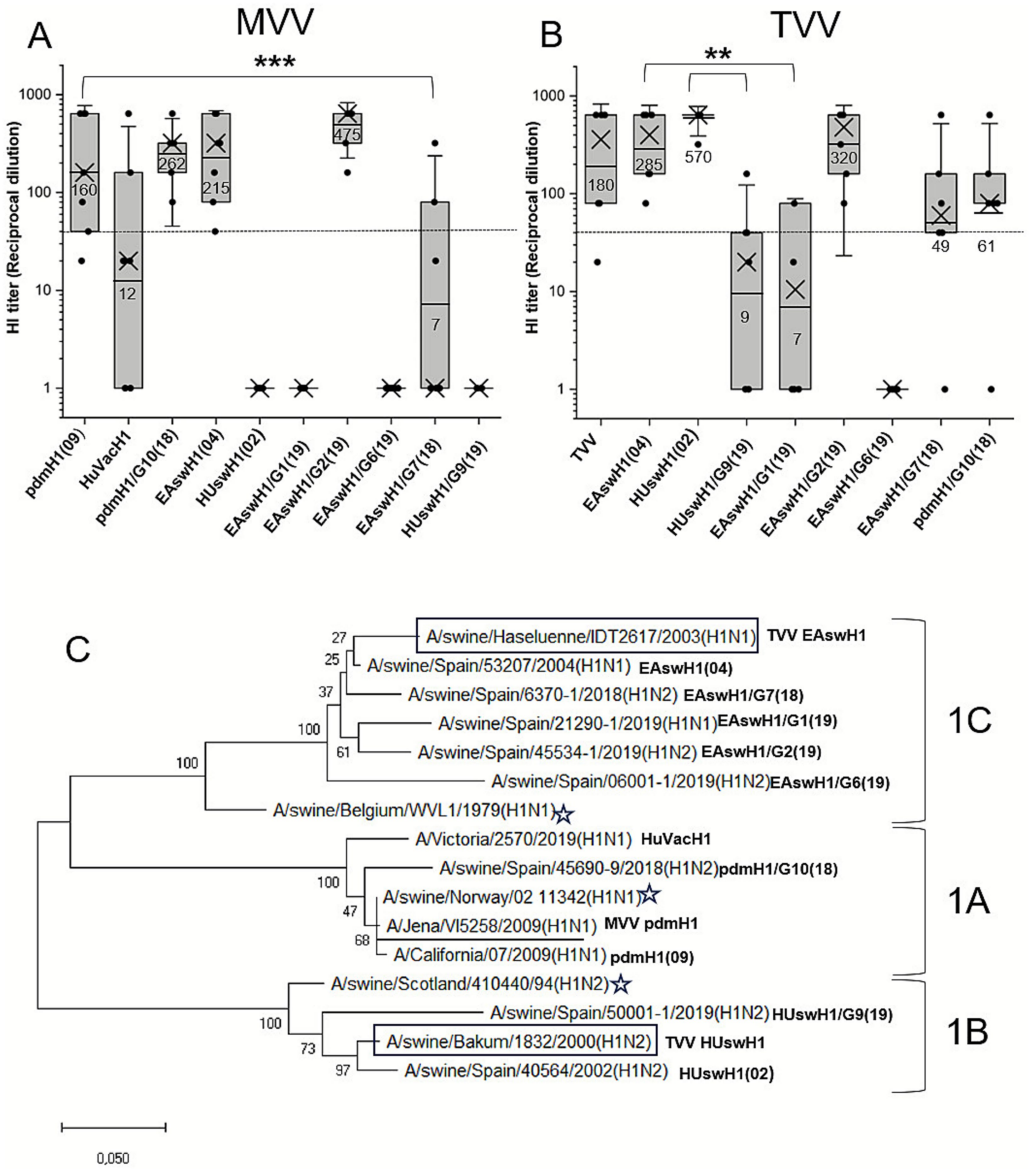
Hemagglutinin inhibition (HI) titers of immunized mice sera against influenza A virus (IAV) hemagglutinin 1 (H1) strains included in this study, and phylogenetic relationship among them. **(A)** HI titers of immunized mice sera with the monovalent swine influenza vaccine (MVV). **(B)**. HI titers of immunized mice sera with the trivalent swine influenza vaccine (TVV). Dots: HI titer of each immunized mouse (*N* = 7). Boxes represent the interquartile range 25–75, horizontal lines and numbers beneath are HI titers geometric means (GM), crosses are median values, and vertical bars represent the standard deviation of the mean. Dashed line is the protection threshold (GM > 40). **(C)** Maximum likelihood phylogenetic tree of predicted protein from IAV H1 strains included in this study. HuVacH1: human seasonal influenza vaccine 2021/2022. H1 lineages are shown in the right side: 1C, Eurasian avian-like H1 (EAswH1), 1B, Human seasonal-like H1 (HUswH1), 1A, pandemic-like H1 (pdmH1). Squared: TVV H1 IAV strains, underlined: MVV H1 strain. Stars: swine IAV clade reference strains. G: genotype of Spanish swine influenza isolates as previously described in Encinas et al. (13).

**Figure 2:**
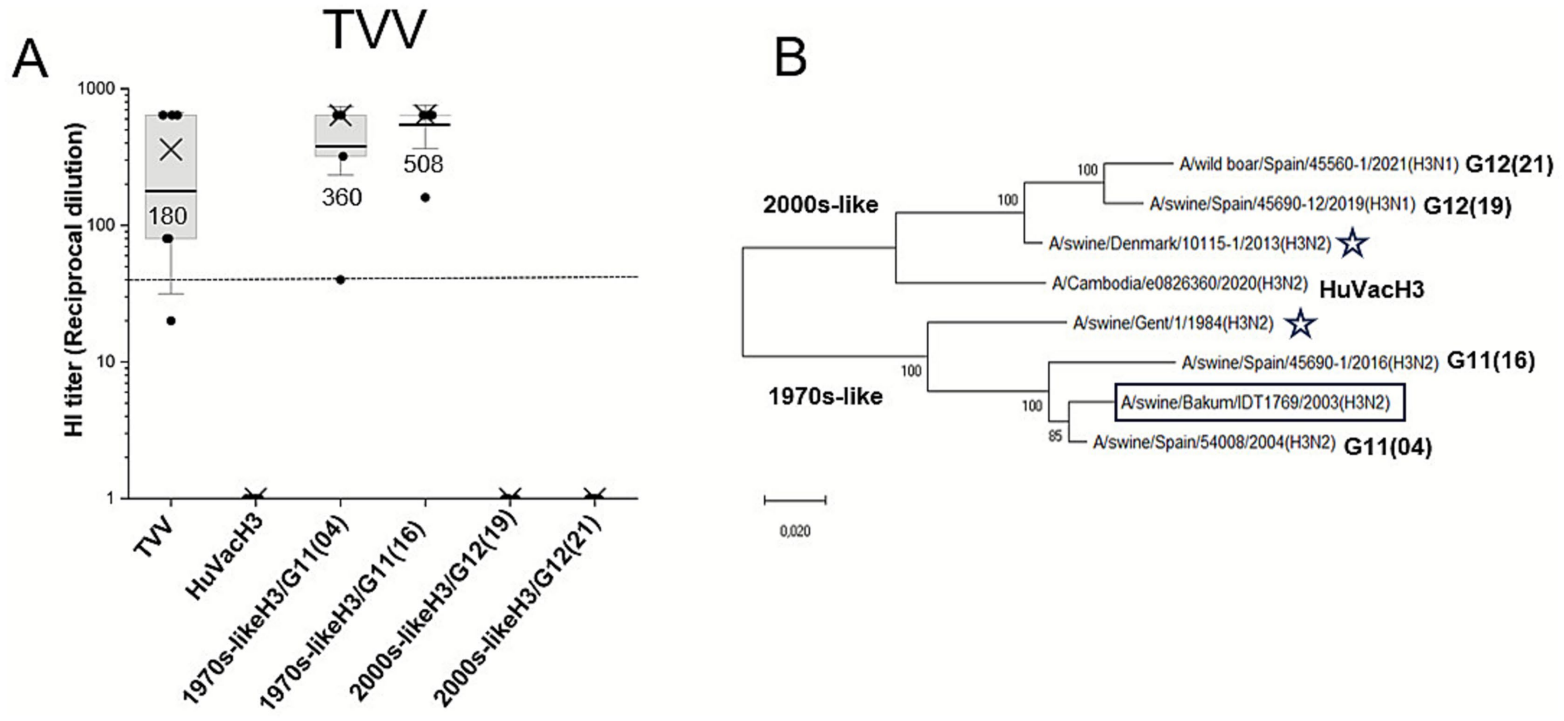
Hemagglutinin inhibition (HI) titers of immunized mice sera against influenza A virus (IAV) hemagglutinin 3 (H3) strains included in this study, and the phylogenetic relationship among them. **(A)**. HI titers of immunized mice sera with the trivalent swine influenza vaccine (TVV). Dots: HI titer of each immunized mouse (*N* = 7). Boxes represent the interquartile range 25–75, horizontal lines and numbers beneath are HI titers geometric means (GM), crosses are median values, and vertical bars represent the standard deviation of the means. Dashed line is the protection threshold (GM > 40). **(B)** Maximum likelihood phylogenetic tree of predicted protein from IAV H3 strains included in this study. HuVacH3: human seasonal influenza vaccine 2021/2022. H3 lineages are shown above/below tree branches: human seasonal-like H3 from the 1970s (1970s-like H3), novel human seasonal-like H3 from the 2000s (2000s-like H3). Squared: TVV H3 IAV strain. Stars: swine IAV clade reference strains. G: genotype of Spanish swine influenza isolates as previously described in Encinas et al. (13).

We previously isolated and identified genetically diverse SIV strains circulating in Spain (2015–2019) through routine surveillance. Influenza-positive samples were isolated and propagated in cell culture, and whole-genome sequenced (Illumina), which led to the description of 12 different genotypes (G), based on their genome sequence, including nine different HA–NA pairings and one internal cassette belonging to a single lineage (EAswH1 or pdmH1), except for the matrix protein segment (13). Nine SIV strains of different genotypes and lineages were selected for assessment: A/swine/Spain/06001-1/2019 (EAswH1/G6(19)), A/swine/Spain/6370-1/2018 (EAswH1/G7(18)), A/swine/Spain/45690-9/2018 (pdmH1/G10(18)), A/swine/Spain/21290-1/2019 (EAswH1/G1(19)), A/swine/Spain/45534-1/2019 (EAswH1/G2(19)), A/swine/Spain/50001-1/2019 (HuswH1/G9(19)), A/wild boar/Spain/45560-1/2021 (2000s.likeH3/G12(21)), A/swine/Spain/45690-12/2019 (2000s.likeH3/G12(19)) and A/swine/Spain/45690-1/2016 (1970s-like H3/G11(16)). Full information on the IAV strains used in this study is available in Table 1. Because homologous vaccine strains were not available, we selected representative SIV strains genetically related to the vaccine strains, as immunization controls: A/California/07/2009 (H1N1, pdmH1(09)) for MVV and A/swine/Spain/54008/2004 (1970s-like H3/G11(04)), A/swine/Spain/53207/2004 (EAswH1(04)) and A/swine/Spain/40564/2002 (HUswH1(02)) for TVV (Table 1). In addition, two human IAV strains from the human seasonal vaccine 2021–2022: A/Cambodia/e0826360/2020 (H3N2, HuVacH3) and A/Victoria/2570/2019 (H1N1, HuVacH1), were included to assess cross-reactivity to swine influenza vaccines (Table 1). Clade assignment of the different isolates was done using the Swine Influenza Global Classification (14) Tool of the Bacterial and Viral Bioinformatics Resource Center (22). To determine the genetic relationship between the vaccine strains and the IAVs used in this assay, protein sequences of hemagglutinins were aligned with ClustalW, and a maximum likelihood tree was constructed with Mega X Jones, using the Taylor–Thornton substitution model with a gamma distribution (Figures 1C, 2B).

**Table 1:** Description of the influenza A viruses (IAVs) used in this study.

SIV study group	Abbreviation	Name	Subtype	Lineage	Clade	Genbank accession number
Vaccine strains	MVV	A/Jena/VI5258/2009	H1N1	H1pdm	1A.3.3.2	KC222636.1
	TVV	Bakum/IDT1769/2003	H3N2	H3	1970.1	GQ161136.1
	TVV	Haselünne/IDT2617/2003	H1N1	EAswH1	1C.2.2	GQ161112.1
	TVV	Bakum/1832/2000 (H1N2)	H1N2	HUswH1	1B.1.2.1	GQ161104.1
Representatives of vaccine strains	MVV, pdmH1(09)	A/California/07/2009	H1N1	H1pdm	1A.3.3.2	NC_026433.1
	TVV, 1970s-like H3/G11(04)	A/swine/Spain/54008/2004	H3N2	HUswH3	1970.1	CY010564.1
	TVV, EAswH1(04)	A/swine/Spain/53207/2004	H1N1	EA_H1	1C.2.1	KR700597.1
	TVV, HUswH1(02)	A/swine/Spain/40564/2002	H1N2	HUswH1	1B.1.2.1	CY116550.1
Spanish field SIV isolates	pdmH1/G10(18)	A/swine/Spain/45690–9/2018	H1N2	H1pdm	1A.3.3.2	MZ945846.1
	EAswH1/G7(18)	A/swine/Spain/6370–1/2018	H1N2	EAswH1	1C.2.1	PQ047764
	EAswH1/G1(19)	A/swine/Spain/21290–1/2019	H1N1	EAswH1	1C.2.1	MZ373100.1
	EAswH1/G6(19)	A/swine/Spain/06001–1/2019	H1N2	EAswH1	1C.2.2	MZ373107.1
	EAswH1/G2(19)	A/swine/Spain/45534–1/2019	H1N2	EAswH1	1C.2.1	MZ945771.1
	HUswH1/G9(19)	A/swine/Spain/50001–1/2019	H1N2	HUswH1	1. B.1.2	MZ945794.1
	2000s-like H3/G12(21)	A/wild boar/Spain/45560–1/2021	H3N1	HUswH3	2000.3	PP338808.1
	2000s-like H3/G12(19)	A/swine/Spain/45690–12/2019	H3N1	HUswH3	2000.3	MZ363769.1
	1970s-like H3/G11(16)	A/swine/Spain/45690–1/2016	H3N2	HUswH3	1970.1	MF872855.1
Human seasonal influenza A 2021/2022 vaccine strains	HuVacH1	A/Victoria/2570/2019	H1N1	H1pdm	1A.3.3.2	OQ719015.1
	HuVacH3	A/Cambodia/e0826360/2020	H3N2	H3_2020		EPI1848532

MVV, monovalent swine influenza vaccine; TVV, trivalent swine influenza vaccine; H1, hemagglutinin 1; H3, hemagglutinin 3; lineage assignment according to Global Swine Influenza Virus Nomenclature system (14); SIV, swine influenza virus; pdmH1, pandemic-like H1; HUswH1, human seasonal-like H1; EAswH1, Eurasian avian-like H1; HUswH3, human seasonal-like H3; HuVac, human seasonal vaccine; G. influenza A virus genotypes previously described (13).

### 2.5 Statistical analysis

Descriptive statistical analysis of the reciprocal dilution HI titers, geometric mean (GM), and median (M) was plotted for each group using Origin2023b. Statistical differences in HI titers between the selected homologous swine influenza vaccine strains and the tested IAV strains were assessed with the Mann–Whitney U-test (*p* < 0.05).

### 2.6 Genomic sequence analysis

IAV HA amino acid sequences were aligned and compared for all the strains used in the HI assay using ClustalW Multiple Alignment (23), and the percentage identity of full-length hemagglutinin (HA) with respect to the vaccine strain was estimated by BioEdit Sequence Alignment Editor and ClustalW software version 7.1. Amino acid substitutions in previously described H1 and H3 antigenic motifs (24–26) were compared among IAV strains. Amino acid numbering was based on the amino acid position on the HA protein, starting with the first methionine.

## 3 Results

### 3.1 Cross-reactive antibodies against H1 SIV strains induced in immunized mice

MVV-immunized mice sera showed HI titers above the threshold (GM > 40) against pdmH1(09), the selected homologous strain, pdmH1/G10(18), and two strains of the Eurasian lineage: EAswH1(04) and EAswH1/G2(19) (Figure 1A). No statistically significant differences were found between these strains. Low levels of cross-reactivity, more than 8-fold reduction with respect to pdmH1(09), were elicited against HuVacH1. No cross-reaction was obtained against SIV of the HUswH1 lineage (Figure 1A, Table 2).

**Table 2:** Antigenic distance among swine influenza virus (SIV) vaccine strains and Spanish field SIV isolates based on the fold change of the hemagglutination inhibition (HI) titers in immunized mice.

Strain name	Clade	A/Jena/VI5258/2009(H1N1)	A/California/07/2009 (pdmH1(09)) ɫ	A/Victoria/2570/2019 (HuVacH1)	A/sw/Spain/45690–9/2018 (pdmH1/G10(18))	A/swine/Bakum/1832/2000(H1N2)	A/swine/Spain/40564/2002 (HuswH1(02)) ɫ	A/sw/Spain/50001–1/2019 (HUswH1/G9(19))	A/swine/Haselünne/IDT2617/2003 (H1N1)	A/swine/Spain/53207/2004 (EAswH1(04)) ɫ	A/swine/Spain/21290–1/2019 (EAswH1/G1(19))	A/swine/Spain/45534–1/2019(EAswH1/G2(19))	A/swine/Spain/06001–1/2019(EAswH1/G6(19))	A/swine/Spain/6370–1/2018(EAswH1/G7(18))	A/swine/Bakum/IDT1769/2003 (H3N2)	A/swine/Spain/54008/2004 (1970s-like H3/G11(04)) ɫ	A/swine/Spain/45690–1/2016 (1970s-like H3/G11(16))	A/Cambodia/e0826360/2020 (HuVacH3)	A/swine/Spain/45690–12/2019(2000s-like H3/G12(19))	A/wild boar/Spain/45560–1/2021 (2000s-like H3/G12(21))
		1A.3.3.2	1A.3.3.2	1A.3.3.2	1A.3.3.2	1B.1.2.1	1B.1.2.1	1B.1.2	1C.2.2	1C.2.1	1C.2.1	1C.2.1	1C.2.2	1C.2.1	1970.1	1970.1	1970.2	2000s	2000.3	2000.3
Monovalent swine influenza vaccine (MVV)																				
A/Jena/VI5258/2009 (H1N1) ¥	1A.3.3.2		**160**	**12**	**262**		**0**	**0**		**215**	**0**	**475**	**0**	**6**						
Trivalent swine influenza vaccine (TVV)																				
A/swine/Bakum/1832/2000(H1N2) ¥	1B.1.2.1						**570**	**9**												
A/swine/Haselünne/IDT2617/2003 (H1N1) ¥	1C.2.2		**61**							**285**	**7**	**320**	**0**	**48**						
A/swine/Bakum/IDT1769/2003 (H3N2) ¥	1970.1															**359**	**507**	**0**	**0**	**0**

¥: strains from the vaccine. ɫ: selected homologous reference strains. Numbers inside the boxes: HI titer geometric mean. Black: > than 8-fold reduction, Dark grey: 4–8-fold reduction, Soft gray: 4-fold reduction, White: equal or increased fold change.

Mice immunized with the TVV showed high HI titers (GM > 250) against EAswH1(04) and HUswH1(02), the selected homologous strains (Figure 1B, Table 2). Furthermore, HI titers above the threshold were found against EAswH1/G2(19), EAswH1/G7(18), and pdmH1/G10(18). However, statistically significant low HI antibody titers were obtained against HUswH1/G9(19), EAswH1/G1(19), and EAswH1/G6(19). Mock vaccinated mice groups were negative for all strains and vaccines.

### 3.2 Cross-reactive antibodies against H3 SIV strains induced in immunized mice with TVV

TVV induced elevated HI titers (GM = 360) against 1970s-like H3/G11(04), the selected homologous strain. This HI titer was even higher (GM = 508) than the recently developed 1970s-like H3/G11(16). However, there was a total lack of cross-reactivity to all swine and human 2000s-like H3 IAV strains tested (Figure 2).

### 3.3 Comparison of HA amino acids among the IAV strains used in this study

**Figure 3:**
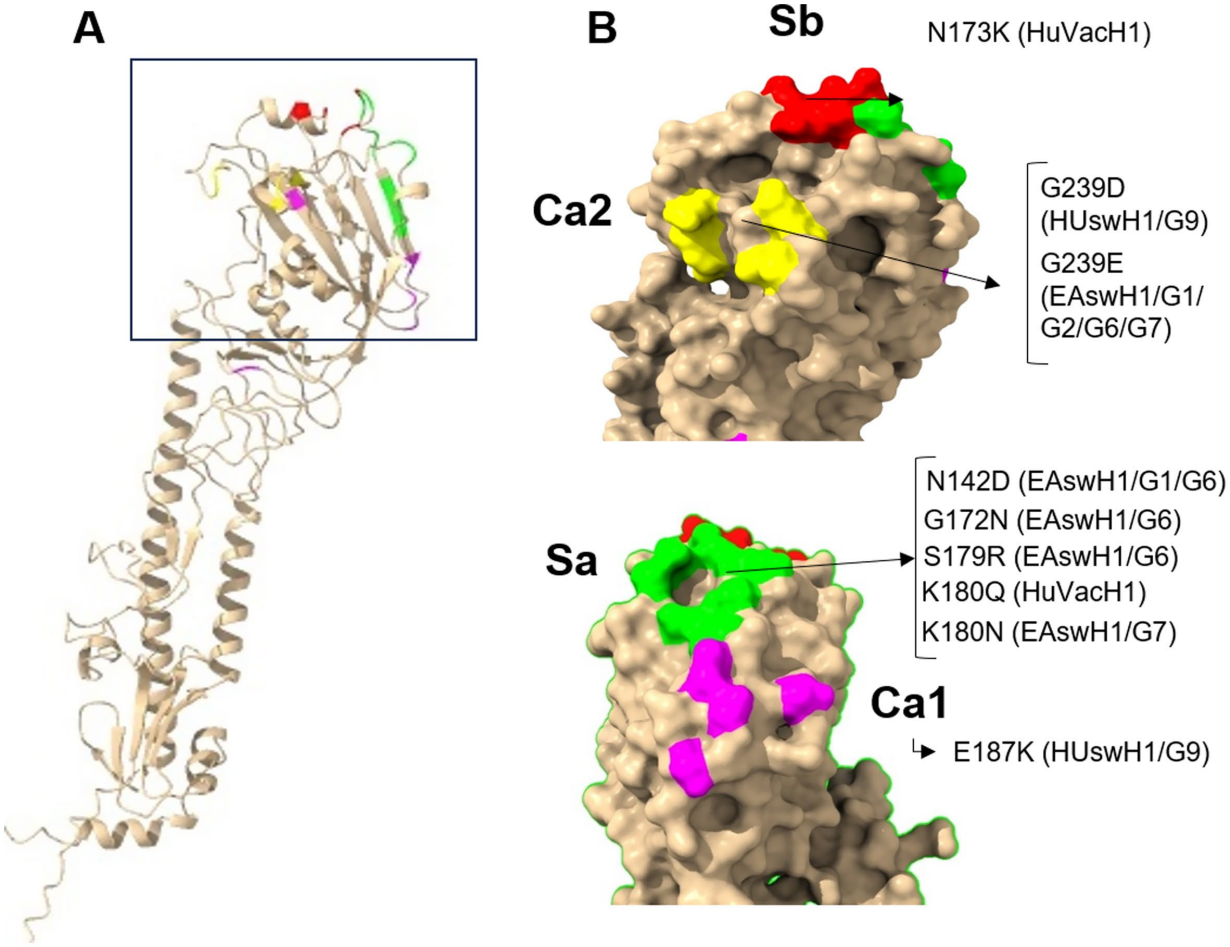
Amino acid substitutions in antigenic motifs of the influenza A virus (IAV) hemagglutinin1 (HA1). **(A)**. Structure of the IAV HA1 protein, squared: head of the HA1. **(B)**. Tridimensional representation of IAV HA1; colored: previously described antigenic motifs in the protein protomer (Ca1, Ca2, Sa, Sb). Amino acid changes in antigenic motifs of swine IAV field isolates with respect to swine IAV vaccine H1 strains; HuVacH1: human seasonal influenza vaccine 2021/2022. HA1 swine European lineages: 1C, Eurasian avian-like H1 (EAswH1), 1B, Human seasonal-like H1 (HUswH1), 1A, pandemic-like H1 (pdmH1). G: genotype of Spanish swine influenza isolates as previously described in Encinas et al. (13). All images were created with UCSF ChimeraX (41).

The selected H1 SIV reference strains showed the highest degree of identity in the full-length HA protein with the corresponding vaccine strain (Table 3) and a close phylogenetic relationship (Figure 1C). Some amino acid changes in H1 antigenic motifs can be observed with respect to the vaccine sequences in each H1 lineage (Figure 3). The pandemic-like lineage showed the lowest degree of variability at the identity level, but HuVacH1 displayed relevant antigenic changes in Sb and Sa motifs with respect to the swine vaccine or SIV isolate. Up to four mutations were found in HUswH1/G9(19) antigenic motifs of the RBS with respect to the vaccine strain. The EAswH1 lineage strains showed the highest degree of variability, with EAswH1/G6(19) being the most different (Table 3, Figure 3).

**Table 3:** Comparison of the amino acid composition of the receptor binding site (RBS) of hemagglutinin 1 (H1) among the swine influenza viruses (SIV) used in this study.

Name and subtype	Clade	%	Antigenic motifs												
			Sa	Ca2	Ca2	Sa	Sb	Sa	Sa	Ca1	Ca1	Ca1	Sb	Sb	Ca2
			142	154	159	172	173	179	180	183	187	196	207	212	239
A/Jena/VI5258/2009 (H1N1)	1A.3.3.2	**MVV**	**N**	**P**	**K**	**G**	**N**	**S**	**K**	**I**	**G**	**I**	**S**	**A**	**D**
A/California/07/2009 (H1N1)	1A.3.3.2	98.9													-
A/sw/Spain/45690–9/2018 (H1N1) G10	1A.3.3.2	95.9		S						V				V	
A/Victoria/2570/2019 (H1N1 HuVacH1)	1A.3.3.2	95.4					K	N	Q						
A/swine/Bakum/1832/2000(H1N2)	1B.1.2.1	**TVV**	**K**	**S**	**S**	**N**	**G**	**S**	**K**	**V**	**E**	**V**	**A**	**E**	**G**
A/swine/Spain/40564/2002 (H1N2)	1B.1.2.1	96.9													N
A/sw/Spain/50001–1/2019 (H1N2) G9	1. B.1.2	90.6								M	K	I			D
A/swine/Haselünne/IDT2617/2003 (H1N1)	1C.2.2	**TVV**	**N**	**S**	**N**	**G**	**N**	**S**	**K**	**T**	**G**	**V**	**T**	**N**	**G**
A/swine/Spain/53207/2004 (H1N1)	1C.2.1	96.4													
A/swine/Spain/6370–1/2018 (H1N2) G7	1C.2.1	94.1							N						E
A/swine/Spain/45534–1/2019 (H1N2) G2	1C.2.1	93.2													E
A/swine/Spain/21290–1/2019 (H1N1) G1	1C.2.1	92.9	D												E
A/swine/Spain/06001–1/2019 (H1N2) G6	1C.2.2	90.6	D			N		R							E

MVV, monovalent swine influenza vaccine; TVV, trivalent swine influenza vaccine. %: Percent identity referring to the full-length H1 with respect to vaccine strains (in bold). Antigenic motifs previously described (24). Antigenic motif numbering is based on the complete H1 sequence from the first methionine. Clade assignment was done according to the global swine influenza nomenclature system (14). Conservative substitutions are shaded light grey; non-conservative substitutions are shaded dark grey. White boxes correspond to identical residues.

The 1970s-like H3 strains showed a percentage of full-length HA protein identity ranging from 97.8% in the selected reference strain to 93.8% in the 2016 strain, with no amino acid changes in antigenic motifs (Table 4). However, the 2000s-like H3 strains displayed substantial differences, as seen in the phylogenetic tree (Figure 2B) and in the numerous amino acid substitutions in HA (Table 4).

**Table 4:** Comparison of the amino acid composition of the receptor binding site (RBS) of hemagglutinin 3 (H3) among the swine influenza viruses (SIV) used in this study.

Name and subtype	Clade	%	Antigenic motifs						
			161	171	172	174	175	205	209
A/swine/Bakum/IDT1769/2003 (H3N2)	1970.1	TVV	N	Y	K	G	N	R	N
A/swine/Spain/54008/2004 (H3N2, 1970s-like H3/G11(04))	1970.1	97.8							
A/swine/Spain/45690–1/2016 (H3N2, 1970s-like H3/G11(16))	1970.1	93.8							
A/Cambodia/e0826360/2020 (H3N2, HuVacH3)	2000.3	82.1	S	T	H	N	Y	K	S
A/swine/Spain/45690–12/2019(H3N1, 2000s-like H3/G12(19))	2000.3	80.5	S	T	H		F	N	S
A/wild boar/Spain/45560–1/2021 (H3N1, 2000s-like H3/G12(21))	2000.3	80	S	T	H		F	N	S
			**145**	**155**	**156**	**158**	**159**	**189**	**193**

TVV, trivalent swine influenza vaccine. %: Percent identity refers to the full-length hemagglutinin (HA) with respect to vaccine strains (in bold). HuVacH3: human seasonal influenza vaccine H3 strain. Antigenic motifs previously described in Souza et al. (26). Antigenic motifs numbering is based on the complete HA sequence from the first methionine; H3 numbering is shown at the bottom of the table (in bold). Clade assignment according to global swine influenza (14). Conservative substitutions are shaded light grey; non-conservative substitutions are shaded dark grey. White boxes correspond to identical residues.

## 4 Discussion

Vaccination is recommended for combating IAV transmission and disease symptoms, but the virus evolves rapidly, requiring periodic strain updates (12). In contrast to humans, there is no formal system for recommending or updating vaccine strains for pigs based on genomic surveillance (27). A rapid way to evaluate the cross-reactive antibody response induced by swine influenza vaccines is to perform *in vitro* or *in vivo* immunological tests against circulating influenza viruses. The mouse is widely used as an animal model for vaccine studies due to its reproducibility, ease of handling, and cost-effectiveness (28). Critically, the mouse model has been validated previously for influenza vaccination results obtained in pigs (29). In this study, we analyzed the cross-reactivity of HI antibodies elicited in mice inoculated with current commercial swine influenza vaccines against field isolates of Spanish SIVs and two representative strains of human IAV circulating in Spain.

The EAswH1/G1(19), EAswH1/G6(19), and HUswH1/G9(19) SIV strains were not recognized by the antibodies induced by the TVV designed to protect against these SIV H1 lineages. Amino acid changes in HA antigenic motifs present in the tested strains relative to the vaccine strain could be responsible for the loss of cross-reactivity, as demonstrated in a previous study comparing the human seasonal H1 vaccine strain and representative pdmH1 SIVs in the United States, where the number of H1 amino acid differences between them was strongly correlated with the antigenic distances estimated by HI assays (30). The amino acid substitutions observed in the Sa motif: N142D, S179R, and K180N may be relevant for antibody recognition, as previously shown in a study aimed at detecting amino acid substitutions that produce escape mutants to monoclonal antibodies targeting pdmH1 HA, where the N142D and K180N substitutions were frequently detected (25). In the case of HUswH1/G9(19) substitutions in Ca1 (E187K) and Ca2 (G239D) may also be responsible for the loss of antibody recognition. It is worth mentioning the marked cross-reactivity observed between heterologous strains. These results, although unexpected, are supported by a previous study in which pigs immunized with a monovalent pdmH1 vaccine showed cross-reactive antibodies against one EAswH1 strain but not against others, and a bivalent EAswH1 vaccine induced cross-reactive antibodies against several pdmH1 strains (31), suggesting that antigenicity relies more on specific amino acids in antigenic motifs than on the whole RBS. Investigating the contribution of each amino acid in the antigenic domain to the antigenicity and specificity of HA for its receptor would be valuable for predicting vaccine suitability.

The high HI titers obtained against the 1970s-like H3 SIV strains contrast with the lack of cross-reactivity obtained against the 2000s-like H3 SIV. The antigenic motifs of 1970s-like H3/G11(16) exactly resemble those present in the RBS of the H3 vaccine strain, so this is not surprising. On the other hand, the 2000s-like H3 SIV strains are quite different from the swine vaccine strain and more similar to the HuVacH3 strain. This H3 lineage carries an HA segment that was introduced from humans into swine in Europe in the 2000s, first detected in Danish pigs (11) and progressively in Spain, Germany, and the Netherlands (32). These strains have undergone less antigenic drift in pig populations than the 1970s-like H3 strains, so they more closely resemble their human ancestor, but are quite different from previous human-origin strains circulating in pigs, showing amino acid replacements in almost all antigenic motifs of the RBS (33). This may be the reason why they are no longer recognized by antibodies induced in vaccinated animals.

In recent years, we have conducted swine influenza surveillance to update our knowledge of the genotypic and phenotypic characteristics of IAV affecting pig herds in Spain and have found a high level of genetic and antigenic variability (13), which raises concerns about the potential emergence of new strains that pose a serious threat to humans and animals. Indeed, swine-derived human influenza variants have recently been found in Europe (34), with some cases in Spain (35). Furthermore, a recently described EAswH1-pdmH1 SIV reassortant in China (36) has been linked to human influenza and increased replication in human cells, raising the possibility of a pandemic. With these premises, we highlight the need to evaluate the efficacy of commercial swine flu vaccines as an essential tool in the fight against influenza viruses.

In Europe, regulations for updating flu vaccines require manufacturers to conduct costly animal trials (37). In contrast, in the United States (US), the Department of Agriculture (USDA) allows assessment of the immunogenicity of additional or updated strains only by serological assays. This allows US vaccine manufacturers to update SIV vaccine strains more flexibly (12). The vaccination rate against swine influenza in Spain is low, probably because in Europe it is not considered a notifiable disease, contrary to avian influenza (38), and the decision to vaccinate is often based on economic interests rather than health measures or because the vaccines are not viewed as effective. The United States also recently licensed a vaccine for SIV that uses an mRNA platform that can more flexibly update vaccine strains to match those in circulation (39). Given that influenza is not only an economic problem for swine producers but a zoonotic risk for humans, Europe must improve monitoring of SIV evolution in its herds and provide more flexibility for updating swine influenza vaccines that prevent the spread.

## Data Availability

The datasets presented in this study can be found in online repositories. The names of the repository/repositories and accession number(s) can be found in the article/supplementary material.

## Author disclaimer

The contributions of the NIH author(s) are considered Works of the United States Government. The findings and conclusions presented in this paper are those of the author(s) and do not necessarily reflect the views of the NIH or the U.S. Department of Health and Human Services, nor imply endorsement by the U.S. Government.

## References

[1] Swine MW. Influenza virus: current status and challenge. Virus Res. (2020) 288:198118. doi: 10.1016/j.virusres.2020.198118, 32798539 PMC7587018

[2] Li Y Robertson I. The epidemiology of Swine influenza. Anim Dis. (2021) 1:21. doi: 10.1186/s44149-021-00024-6, 34778883 PMC8476212

[3] Van Reeth K Vincent AL. Influenza viruses. In: Zimmerman JJ, Karriker LA, Ramirez A, Schwartz KJ, Stevenson GW, Zhang j, editors. Diseases of Swine. (2019). p. 576–93. doi: 10.1002/9781119350927.ch36

[4] Hervé S Rose N Barbier N Quéguiner S Gorin S Fonseca R . Trends in Seroprevalence of influenza a virus infections in pigs in France (2008-2022). Porcine Health Manag. (2025) 11:42. doi: 10.1186/s40813-025-00455-4, 40722119 PMC12306050

[5] Szablewski CM McBride DS Trock SC Habing GG Hoet AE Nelson SW . Evolution of influenza a viruses in exhibition Swine and transmission to humans, 2013-2015. Zoonoses Public Health. (2024) 71:281–93. doi: 10.1111/zph.13104, 38110691 PMC10994755

[6] Lee CY. Exploring potential intermediates in the cross-species transmission of influenza a virus to humans. Viruses. (2024) 16:29. doi: 10.3390/v16071129, 39066291 PMC11281536

[7] Nelson MI Stratton J Killian ML Janas-Martindale A Vincent AL. Continual reintroduction of human pandemic H1n1 influenza a viruses into Swine in the United States, 2009 to 2014. J Virol. (2015) 89:6218–26. doi: 10.1128/JVI.00459-15, 25833052 PMC4474294

[8] Nelson MI Viboud C Vincent AL Culhane MR Detmer SE Wentworth DE . Global migration of influenza a viruses in Swine. Nat Commun. (2015) 6:6696. doi: 10.1038/ncomms7696, 25813399 PMC4380236

[9] Nelson MI Vincent AL. Reverse zoonosis of influenza to Swine: new perspectives on the human-animal Interface. Trends Microbiol. (2015) 23:142–53. doi: 10.1016/j.tim.2014.12.002, 25564096 PMC4348213

[10] Lorbach JN Nelson SW Lauterbach SE Nolting JM Kenah E McBride DS . Influenza vaccination of Swine reduces public health risk at the Swine-human Interface. mSphere. (2021) 6:e0117020. doi: 10.1128/mSphere.01170-20 PMC8265676 34190586

[11] Anderson TK Chang J Arendsee ZW Venkatesh D Souza CK Kimble JB . Swine influenza a viruses and the tangled relationship with humans. Cold Spring Harb Perspect Med. (2021) 11:737. doi: 10.1101/cshperspect.a038737, 31988203 PMC7919397

[12] Mancera Gracia JC Pearce DS Masic A Balasch M. Influenza a virus in Swine: epidemiology, challenges and vaccination strategies. *Frontiers in veterinary*. Science. (2020) 7:7. doi: 10.3389/fvets.2020.00647, 33195504 PMC7536279

[13] Encinas P Del Real G Dutta J Khan Z van Bakel H Del Burgo MAM . Evolution of influenza a virus in intensive and free-range Swine farms in Spain. Virus Evol. (2022) 7:veab099. doi: 10.1093/ve/veab099, 35039784 PMC8754697

[14] Anderson TK Macken CA Lewis NS Scheuermann RH Van Reeth K Brown IH . A phylogeny-based global nomenclature system and automated annotation tool for H1 hemagglutinin genes from Swine influenza a viruses. mSphere. (2016) 1:16. doi: 10.1128/mSphere.00275-16 PMC5156671 27981236

[15] Petro-Turnquist E Pekarek MJ Weaver EA. Swine influenza a virus: challenges and novel vaccine strategies. Front Cell Infect Microbiol. (2024) 14:1336013. doi: 10.3389/fcimb.2024.1336013, 38633745 PMC11021629

[16] Lim CML Komarasamy TV Adnan N Radhakrishnan AK Balasubramaniam V. Recent advances, approaches and challenges in the development of universal influenza vaccines. Influenza Other Respir Viruses. (2024) 18:e13276. doi: 10.1111/irv.13276, 38513364 PMC10957243

[17] Vincent AL Perez DR Rajao D Anderson TK Abente EJ Walia RR . Influenza a virus vaccines for Swine. Vet Microbiol. (2017) 206:35–44. doi: 10.1016/j.vetmic.2016.11.026, 27923501 PMC8609643

[18] World Health Organization In: World Health Organization, editor. Tool for influenza pandemic risk assessment (Tipra). Geneva: (2016)

[19] Black S Nicolay U Vesikari T Knuf M Del Giudice G Della Cioppa G . Hemagglutination inhibition antibody titers as a correlate of protection for inactivated influenza vaccines in children. Pediatr Infect Dis J. (2011) 30:1081–5. doi: 10.1097/INF.0b013e3182367662, 21983214

[20] Gonzalo RM del Real G Rodriguez JR Rodriguez D Heljasvaara R Lucas P . A heterologous prime-boost regime using DNA and recombinant vaccinia virus expressing the Leishmania Infantum P36/lack antigen protects Balb/C mice from cutaneous Leishmaniasis. Vaccine. (2002) 20:1226–31. doi: 10.1016/s0264-410x(01)00427-3, 11803085

[21] World Health Organization. Manual for the laboratory diagnosis and Virological surveillance of influenza. Geneva: World Health Organization (2011).

[22] Olson RD Assaf R Brettin T Conrad N Cucinell C Davis JJ . Introducing the bacterial and viral bioinformatics resource center (Bv-Brc): a resource combining Patric, Ird and Vipr. Nucleic Acids Res. (2023) 51:D678–d89. doi: 10.1093/nar/gkac1003 36350631 PMC9825582

[23] Thompson JD Higgins DG Gibson TJ. Clustal W: improving the sensitivity of progressive multiple sequence alignment through sequence weighting, position-specific gap penalties and weight matrix choice. Nucleic Acids Res. (1994) 22:4673–80. doi: 10.1093/nar/22.22.4673, 7984417 PMC308517

[24] Caton AJ Brownlee GG Yewdell JW Gerhard W. The antigenic structure of the influenza virus a/Pr/8/34 hemagglutinin (H1 subtype). Cell. (1982) 31:417–27. doi: 10.1016/0092-8674(82)90135-0, 6186384

[25] Matsuzaki Y Sugawara K Nakauchi M Takahashi Y Onodera T Tsunetsugu-Yokota Y . Epitope mapping of the hemagglutinin molecule of a/(H1n1)Pdm09 influenza virus by using monoclonal antibody escape mutants. J Virol. (2014) 88:12364–73. doi: 10.1128/jvi.01381-14, 25122788 PMC4248900

[26] Souza CK Anderson TK Chang J Venkatesh D Lewis NS Pekosz A . Antigenic distance between north American Swine and human seasonal H3n2 influenza a viruses as an indication of zoonotic risk to humans. J Virol. (2022) 96:e0137421. doi: 10.1128/jvi.01374-21, 34757846 PMC8791259

[27] Reeth KV Vincent AL Lager KM. Vaccines and vaccination for Swine influenza: differing situations in Europe and the USA. In: Swayne DE, editor. Animal Influenza. (2016). 480–501. doi: 10.1002/9781118924341.ch19

[28] Warang P Singh G Moshir M Binazon O Laghlali G Chang LA . Impact of Fcrn antagonism on vaccine-induced protective immune responses against viral challenge in Covid-19 and influenza mouse vaccination models. Hum Vaccin Immunother. (2025) 21:2470542. doi: 10.1080/21645515.2025.2470542, 40028815 PMC11881870

[29] Petro-Turnquist EM Madapong A Pekarek M Steffen D Weaver EA. Epitope-optimized vaccine elicits enduring immunity against Swine influenza a virus. Nat Commun. (2025) 16:4046. doi: 10.1038/s41467-025-59182-7, 40301303 PMC12041224

[30] Ciacci Zanella G Markin A Neveau Thomas M Snyder CA Souza CK Arruda B . Transmission and pathologic findings of divergent human seasonal H1n1pdm09 influenza a viruses following spillover into pigs in the United States. Influenza Other Respir Viruses. (2025) 19:e70128. doi: 10.1111/irv.70128, 40671507 PMC12268112

[31] Parys A Vandoorn E Chiers K Van Reeth K. Alternating 3 different influenza vaccines for swine in Europe for a broader antibody response and protection. Vet Res. (2022) 53:44. doi: 10.1186/s13567-022-01060-x, 35705993 PMC9202218

[32] Coggon A Lopes S Simon G Arendsee Z Chen KF Chiapponi C . Quantifying the zoonotic risk profile of European influenza a viruses in Swine from 2010 to 2020 inclusive. J Virol. (2025) 99:e0030625. doi: 10.1128/jvi.00306-25, 40464577 PMC12288490

[33] Koel BF Burke DF Bestebroer TM van der Vliet S Zondag GC Vervaet G . Substitutions near the receptor binding site determine major antigenic change during influenza virus evolution. Science. (2013) 342:976–9. doi: 10.1126/science.1244730 24264991

[34] ECDC. Zoonotic Influenza. Annual Epidemiological Report 2022. Stockholm: European Centre for Disease Prevention and Control.

[35] World Health Organization. Influenza a(H1n1) Variant Virus - Spain (2024). Available online at: https://www.who.int/emergencies/disease-outbreak-news/item/2024-DON503 (Accessed October 5, 2025).

[36] Sun H Xiao Y Liu J Wang D Li F Wang C . Prevalent Eurasian avian-like H1n1 Swine influenza virus with 2009 pandemic viral genes facilitating human infection. Proc Natl Acad Sci. (2020) 117:17204–10. doi: 10.1073/pnas.1921186117, 32601207 PMC7382246

[37] Regulation (EU) 2019/6 of the European Parliament and of the Council of 11 December 2018 on veterinary medicinal products and repealing Directive 2001/82/EC, (7 January 2019), Official Journal of the European Union, L4, p. 43–167. Available online at: https://eur-lex.europa.eu/eli/reg/2019/6/oj/eng

[38] Regulation (EU) 2016/429 of the European Parliament and of the Council of 9 March 2016 on transmissible animal diseases and amending and repealing certain acts in the area of animal health (´Animal Health Law´),(31 March 2016), Official Journal of the European Union, L 84, 59. Available online at: https://eur-lex.europa.eu/legal-content/EN/TXT/HTML/?uri=OJ:L:2016:084:FULL

[39] Merck Animal Health. Swine vaccine platform Sequivity. Available online at: https://www.merck-animal-health-usa.com/species/swine/sequivity (Accessed October 5, 2025).

[40] Encinas P Nogales A Nelson M García-Sastre A Del Real G. Efficacy of current swine influenza vaccine in Spain. Authorea [Preprint]. (2025). doi: 10.22541/au.173876764.41253945/v1

[41] Petterson EF Goddard TD Huang CC Meng EC Couch GS Croll TI . UCSF ChimeraX: Structure visualization for researchers, educators, and developers. Protein Sci. (2021) 30:70–82. doi: 10.1002/pro.3943 32881101 PMC7737788

